# Enhancing psychological performance and basketball skills: a comparative study of elite athletes and college recreational players after an 8-week mindfulness intervention

**DOI:** 10.3389/fpsyg.2026.1794656

**Published:** 2026-04-22

**Authors:** Qi-Fan Wu, Jia-Qi Du

**Affiliations:** College of Physical Education, Anhui Normal University, WuHu, China

**Keywords:** basketball skills, comparative study, mental training, mindfulness intervention, psychological performance

## Abstract

**Background:**

In high-intensity sports such as basketball, psychological factors significantly influence performance. Mindfulness-based interventions (MBIs) show potential for reducing anxiety, depression, and mental fatigue while enhancing attention, resilience, and basketball technical skills. However, few studies have compared the effects of MBI between elite athletes and recreational players.

**Purpose:**

This research examined the effects of an 8-week Mindfulness Acceptance Insight Commitment (MAIC) intervention on psychological performance and technical skills among elite basketball athletes and collegiate recreational players, while investigating skill level as a moderator.

**Methods:**

Sixty seven participants (24 elite athletes, 43 recreational players) were randomly assigned to experimental or control groups. The MAIC program consisted of two 60-min sessions per week. Psychological measures included the State–Trait Anxiety Inventory (STAI), Beck Depression Inventory-II (BDI-II), Schultz Attention Test (SAT), and Conner-Davidson Resilience Scale (CD-RISC). Basketball skill assessments comprised the Speed and Accurate Shooting Test, Obstacle Dribbling Test, Defensive Sliding Test, Lay-up Test, and No-Ball Maneuver Running. Assessments were conducted pre- and post-intervention.

**Results:**

Sixty three participants completed the study. Elite athletes showed significant improvements in STAI, CD-RISC, and Obstacle dribbling test performance post-MAIC, with a trend toward benefit in the SAT. Recreational players demonstrated broader benefits, including improvements in Speed and Accurate Shooting Test, Lay-up Test, STAI, SAT, and CD-RISC. ANOVA revealed that, controlling for baseline measures, recreational basketball players demonstrated greater benefits than elite athletes in shooting, dribbling, anxiety, and depression, indicating a skill-level moderating effect.

**Conclusion:**

MAIC enhances psychological and technical performance differentially: elite athletes primarily benefit in targeted psychological domains, while recreational players experience more comprehensive psychological and skill-related effects. These findings support personalized mindfulness interventions in basketball, enriching sports psychology theory and practice.

## 1 Introduction

Over the past decade, competitive sports have become more intense, making the effective enhancement of athletic performance a key focus among coaches, practitioners, and athletes. Within this field, the critical role of psychological factors in athletic performance has become increasingly recognized, particularly in high-intensity sports involving extensive decision-making like basketball. High-level athletes frequently face intense competitive pressures, injury risks, and the burden of meeting external expectations. These pressures often lead to symptoms such as anxiety, depression, distractibility, and reduced psychological resilience (Bu et al., 2020). Ultimately, these symptoms can diminish both mental health and skill execution (Bühlmayer et al., 2017; Si et al., 2024; Simpson et al., 2020). Traditional psychological skill training (PST) has been extensively developed and applied to alleviate psychological issues, including goal setting, imagery training, and progressive relaxation techniques (Noetel et al., 2019). However, numerous randomized controlled trials and meta-analyses have shown that MBIs offer athletes an approach that complements cognitive behavioral training and may yield superior results (Kanaujia et al., 2023; Koop and Jooste, 2023; Wang et al., 2023b). By cultivating present-moment awareness, emotional acceptance, and cognitive flexibility, mindfulness not only significantly reduces performance anxiety and negative emotions but also enhances flow states, attentional regulation, and overall mental health (Ptáček et al., 2024; Yang et al., 2025).

Originating in Eastern Zen teachings, mindfulness is academically defined as nonjudgmental awareness of the present moment. In sports psychology, it is primarily applied through the Mindfulness-Acceptance-Commitment (MAC) framework, the Mindfulness-Based Sports Performance Enhancement (MSPE) program, and Mindfulness-Based Stress Reduction (MBSR) (Gardner and Moore, 2007; Kaufman et al., 2009). Meta-analyses indicate that mindfulness interventions significantly enhance athletes’ mindfulness levels and sports performance. Concurrently, mindfulness practice effectively reduces athletes’ anxiety, depression, and psychological fatigue (Si et al., 2024; Wang et al., 2023a). These improvements in psychological indicators are crucial for competitive stress management and athletic performance under high-pressure conditions. The mechanism linking psychological performance and athletic skill can be explained by embodied cognition theory (Foglia and Wilson, 2013), which posits that cognition is embodied, embedded in the environment, and action-oriented. Athletes’ mental health and performance form a positive feedback loop through the body, where negative or positive mental states directly translate into tense or fluid physical performance (Beilock, 2008). Enhanced psychological components bolster emotional regulation, attentional control, and resilience, enabling athletes to adapt to uncertainties and setbacks during competition (Khoeini, 2026).

Basketball demands rapid decision-making, precise motor skills, and sustained focus, making mindfulness interventions particularly promising for this sport. Extensive empirical research demonstrate that mindfulness training significantly enhances focus and emotional stability during critical game moments, while also improving free throw and three-point shooting performance, execution of complex tactics, and overall technical skill performance (Goisbault et al., 2022; Kanaujia et al., 2023; Wang and Zhang, 2025). Further research indicates that brief 15- to 30-min mindfulness interventions can alleviate mental fatigue in basketball players, restore attention levels, and enhance decision-making and tactical performance during small-court scrimmages. Additionally, mindfulness correlates with reduced mental fatigue, improved recovery, and optimized heart rate variability during basketball-specific tasks, indirectly promoting offensive and defensive capabilities (Perry et al., 2017).

Although the efficacy of mindfulness interventions has been well-documented, varying technical levels across different populations may influence outcomes. Meta-analyses indicate that lower-level or recreational athletes often exhibit greater skill improvements, potentially due to lower baseline psychological performance, whereas elite athletes frequently experience a ceiling effect (Wang et al., 2023a; Yang et al., 2025). High-level athletes, possessing greater baseline psychological resilience and skill proficiency, may gain more targeted benefits in areas like attention and anxiety management. Recreational athletes, conversely, benefit more broadly from improved overall mental health, reduced negative emotions, and foundational skill acquisition (Tierney, 2020; Wang et al., 2023a). College basketball enthusiasts who are not majoring in sports, as a typical recreational-level group, offer high accessibility and representativeness, facilitating recruitment and enabling effective comparisons of intervention differences across athletes of varying skill levels. However, no studies have directly compared elite and recreational athletes within the same randomized controlled design, particularly in basketball. Existing research also focuses on testing single groups or metrics, lacking comprehensive examination of psychological indicators and specific skills.

This randomized controlled trial examined the effects of an 8-week mindfulness intervention on psychological indicators (anxiety, depression, attention, resilience) and basketball-specific skills (shooting, dribbling, defensive sliding, agility drills, layups) among elite basketball athletes and recreational college players. It further investigated whether skill level moderates these effects. Specifically, we hypothesized that (1) mindfulness intervention can effectively enhance basketball skills and psychological performance in both recreational and elite players. (2) Recreational players would demonstrate greater psychological and skill improvements following the mindfulness intervention.

## 2 Methods

### 2.1 Participants

**Figure 1:**
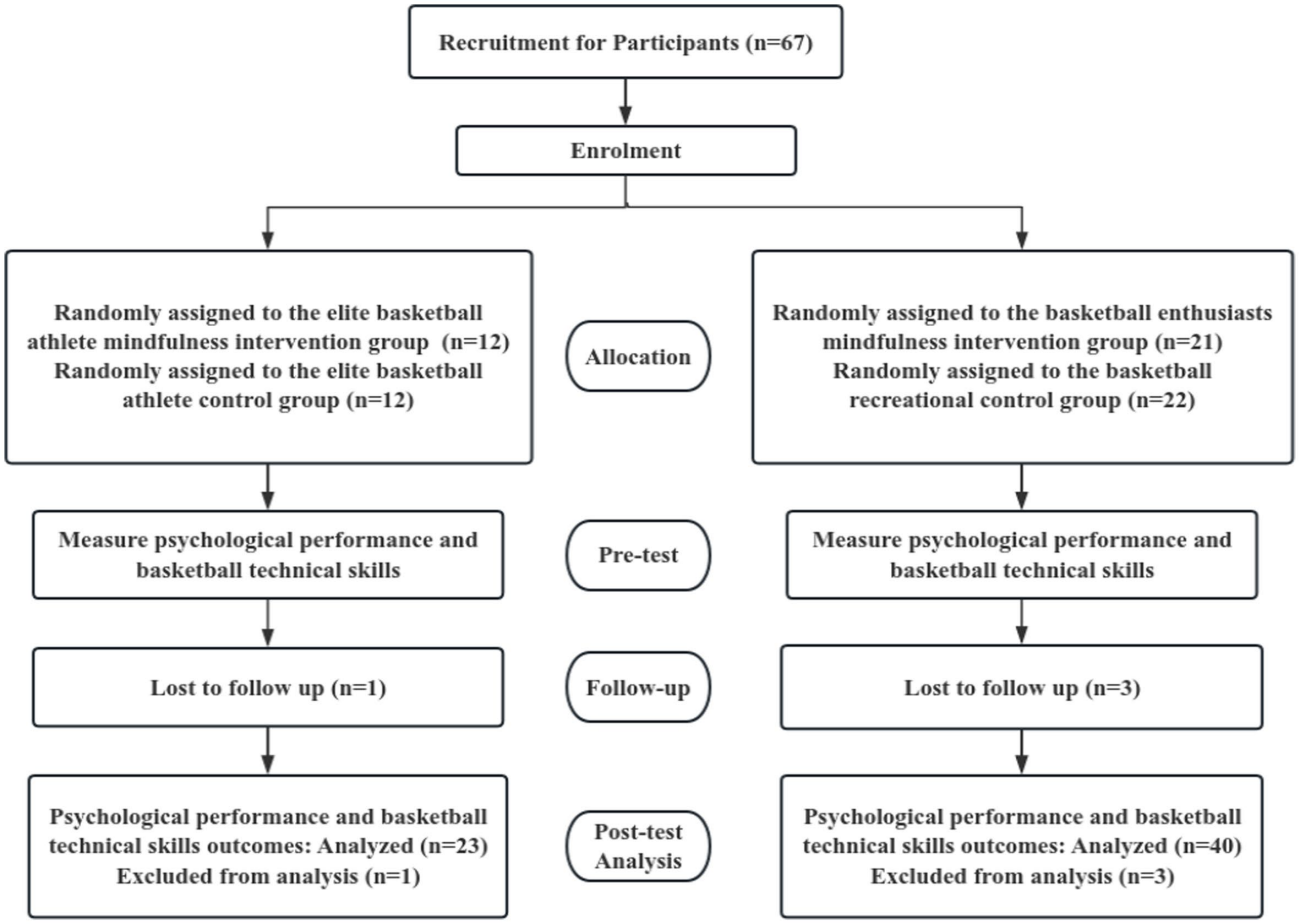
Recruitment process consort flowchart. Source: https://www.consort-spirit.org/

Participants in this study were recruited from local basketball associations, university basketball teams, and college students. Inclusion/exclusion/dropout criteria are detailed in Table 1. A total of 67 individuals were recruited, comprising 24 elite basketball athletes and 43 recreational basketball players. Randomization was conducted using a computer-generated random number sequence. Participants within each stratum (elite athletes and recreational players) were assigned to the experimental or control group using block randomization with a fixed block size of four, ensuring balanced group allocation. Group assignments were concealed in sealed opaque envelopes and disclosed only after baseline assessments were completed, thereby ensuring allocation concealment. All participants completed data collection prior to the intervention. During the intervention, one elite athlete (*n* = 1) and three recreational players (*n* = 3) requested to discontinue the experimental intervention. Baseline data from participants who did not complete the study were also excluded from the data analysis. Ultimately, 63 participants completed the entire experiment, comprising the elite mindfulness experimental group (*n* = 12), elite control group (*n* = 11), recreational mindfulness experimental group (*n* = 20), and recreational control group (*n* = 20). The detailed CONSORT recruitment flowchart is shown in Figure 1. A sensitivity analysis was conducted using G*Power 3.1.9.7 to determine the minimum effect size detectable with the present sample (*n* = 63), assuming *α* = 0.05 and power (1−*β*) = 0.80. Results indicated that the study was adequately powered to detect medium-to-large effect sizes (*f* ≥ 0.35), consistent with effect sizes reported in comparable mindfulness intervention studies (Cioffi et al., 2025).

**Table 1:** Inclusion/exclusion/dropout criteria.

	Elite basketball players	Recreational basketball players
Inclusion criteria	1. National level basketball player; 2. Engage in ≥10 h of basketball training per week for ≥5 consecutive years; 3. No major injuries or illnesses within the past 6 months; 4. Voluntarily participate and sign an informed consent form.	1. Participate in basketball activities (training or games) for at least 4 h per week; 2. No major injuries or illnesses in the past 6 months; 3. Volunteer participation and signed informed consent.
Exclusion criteria	1. Have undergone mindfulness, meditation, or similar psychological intervention training within the past 6 months; 2. Are currently taking medications that affect mental state or cognitive function (e.g., antidepressants, anti-anxiety drugs); 3. Have severe cardiovascular disease, musculoskeletal disorders, or other conditions unsuitable for high-intensity basketball skill testing; 4. Are unable to complete the 8-week intervention or pre- and post-tests (due to academic commitments, injury, illness, or other reasons).	
Dropout criteria	1. Intervention course attendance rate below 70%; 2. Voluntary withdrawal from the study due to injury, illness, academic commitments, or other personal reasons; 3. Significant life events occurring during the intervention period that may impact psychological or skill performance (e.g., serious injury, family crisis), and which researchers assess as potentially interfering with study outcomes.	

### 2.2 Mindfulness intervention protocol

The mindfulness intervention in this study was implemented at the Sports Laboratory of Anhui Normal University, involving an 8-week systematic training program for participants in the mindfulness group. Sessions were conducted twice weekly, each lasting 60 min. The intervention employed the Mindfulness-Acceptance-Insight-Commitment (MAIC) training program (Su et al., 2019). MAIC was specifically designed for Chinese athletes and exhibits high cultural adaptability. Developed by Si et al. (2014) based on the Western MAC framework, its core strength lies in its localized design. MAIC fully integrates the mindset, training habits, and cultural-psychological characteristics of Chinese athletes, making it more easily understood and accepted. Furthermore, the program has been widely applied across various sports disciplines in mainland China and Hong Kong, including shooting, tennis, and synchronized swimming (Su et al., 2019). Research indicates MAIC is highly compatible with psychological interventions for athletes within the Chinese cultural context and effectively mitigates intervention efficacy decline caused by cultural differences (Bu et al., 2020; Feng and Si, 2015). This is crucial for the present study, as the sample primarily consisted of Chinese university students and elite basketball athletes. MAIC’s focus on enhancing athletic performance aligns closely with the objectives of this research. Related studies (Bu et al., 2020; Feng and Si, 2015; Su et al., 2019) confirm that MAIC significantly improves athletes’ concentration, emotional regulation, and overall athletic performance—core factors influencing the psychological and skill metrics examined in this study. The specific mindfulness intervention protocol is detailed in Supplementary File 1.

### 2.3 Psychological performance metrics

#### 2.3.1 Anxiety

Anxiety levels were measured using the Chinese version of the State–Trait Anxiety Inventory (STAI). Developed by Spielberger (1983), this scale comprises two subscales: The State Anxiety subscale (S-Anxiety, 20 items, assessing momentary anxiety) and the Trait Anxiety subscale (T-Anxiety, 20 items, assessing general anxiety tendency). Each item uses a 4-point Likert scale (1 = Almost never, 4 = Very often), with completion typically taking 15–20 min. The total score ranges 20–80, with higher scores indicating greater anxiety levels. Previous studies demonstrate excellent reliability and validity (Cronbach’s *α* > 0.90), and the Chinese version has been validated across diverse populations (Du et al., 2022; Lyu et al., 2022; Tang et al., 2023).

#### 2.3.2 Depression

The Beck Depression Inventory-Second Edition (BDI-II) assesses depressive levels. Revised by Beck et al. (1996), it comprises 21 item groups, each containing four statements rated on a 0–3 severity scale. Participants were asked to complete the self-report independently, which typically takes 5 to 15 min. The scale covers cognitive, emotional, physical, and motivational symptoms. Total scores range from 0 to 63 (0–13: no depression; 14–19: mild; 20–8: moderate; 29–63: severe). The Chinese version of the BDI-II has demonstrated good reliability and validity among Chinese college students and athletes (Cronbach’s *α* > 0.85) (Liao et al., 2017; Wang and Gorenstein, 2013; Yeung et al., 2002).

#### 2.3.3 Schultz attention test (SAT)

The test material consisted of a 3 × 3 grid containing randomly arranged numbers 1–9. Participants were required to locate and verbally report each number in ascending order as quickly as possible. Upon an incorrect location or verbal report, the system immediately emitted an auditory cue (500 milliseconds, 1,000 Hz), which required the participants to relocate the correct number. After completing one sequence, the next sequence began. Each participant completed three consecutive full test sets, each using a different randomized grid layout. The arithmetic mean of the three test durations served as the final indicator of the participants’ attention shifting and attention transition abilities. Shorter durations indicated higher attention search efficiency, greater flexibility in attention shifting, and more stable attention transition processes. The SAT originated from the Schultz Grid Training Method; it reflects a participants’ level of attention by recording the time taken to complete tasks and has broad applicability (Lu et al., 2022).

#### 2.3.4 Resilience

Psychological resilience was assessed using the 25-item Connor-Davidson Resilience Scale (CD-RISC). This scale encompassed five dimensions: personal competence, stress tolerance, positive acceptance of change, sense of control, and spiritual influence (Connor and Davidson, 2003). Test completion typically takes 5–10 min, with total scores ranging from 0 to 100, where higher scores indicate greater resilience. The Chinese version of the CD-RISC has demonstrated excellent reliability and validity (Cronbach’s *α* > 0.90) among Chinese athletes and university students (Liu and Wang, 2025; Yu and Zhang, 2007).

### 2.4 Basketball technical skill indicators

Basketball technical skill metrics primarily encompass shooting, dribbling, shuffling, layups, and off-ball movement components. The specific implementation of the testing was adapted from established research paradigms from previous studies (Apostolidis et al., 2004; Bös et al., 1987; Getchell, 1979; Hopkins et al., 1984; Zacharakis et al., 2020). Basketball technical skill metrics primarily consist of shooting accuracy under time pressure, dribbling decision-making speed, zone movement efficiency, lateral movement speed, lower-body coordination, ability to execute sudden stops and pivots, and postural control.

#### 2.4.1 Speed and accuracy shooting test (SAST)

**Figure 2:**
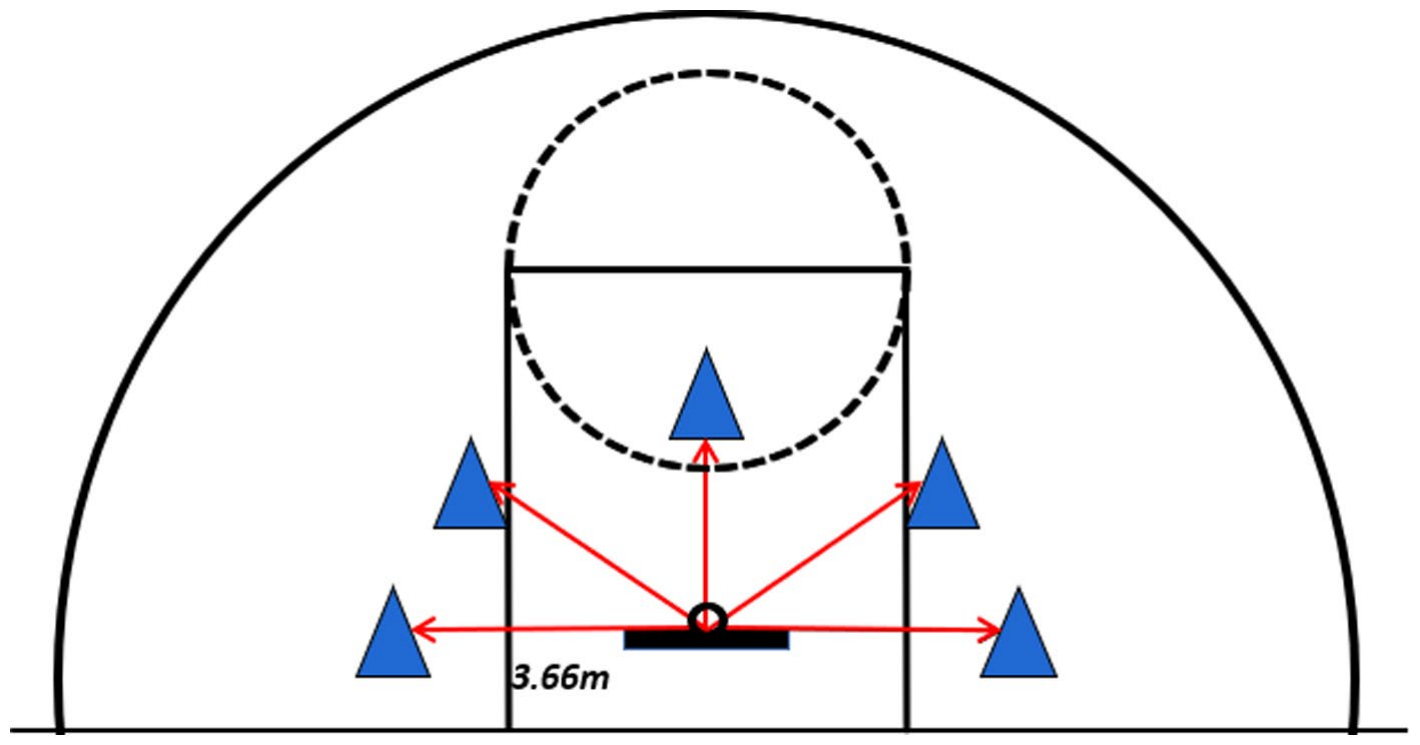
Speed and accurate shooting test.

All participants completed three trials, with the first serving as a practice and familiarization phase, followed by two timed trials. A 3-min rest period was provided between each trial. The testing area was positioned behind the standard free-throw line, where participants stood in a marked zone 12 feet (3.66 m) from the backboard. Upon hearing the “Ready—Go” command, participants immediately began shooting and were required to retrieve the rebound themselves, dribble to another designated area, and shoot again. No more than four dribbles were permitted between shots, and consecutive dribbles were prohibited. Each shot originated from a different designated area. Violations, such as traveling or double dribbling, resulted in the shot being disallowed. If two consecutive dribbles occurred, the subsequent shot was scored as 0 points even if successful. Similarly, if more than four dribbles were taken for a single shot, the attempt was recorded as 0 points. Instances where participants failed to complete shots from all five designated zones were rendered invalid, requiring a retake of the test (Figure 2). An assistant coach recorded the designated area for each shot, the number of dribbles, and any violations. Scoring was defined as follows: 2 points for a made shot, 1 point for a shot that touched the rim but did not enter, and 0 points for a shot that missed the rim entirely. The final score consisted of the sum of the scores from the two 60-s official trials.

#### 2.4.2 Obstacle dribbling test (ODT)

**Figure 3:**
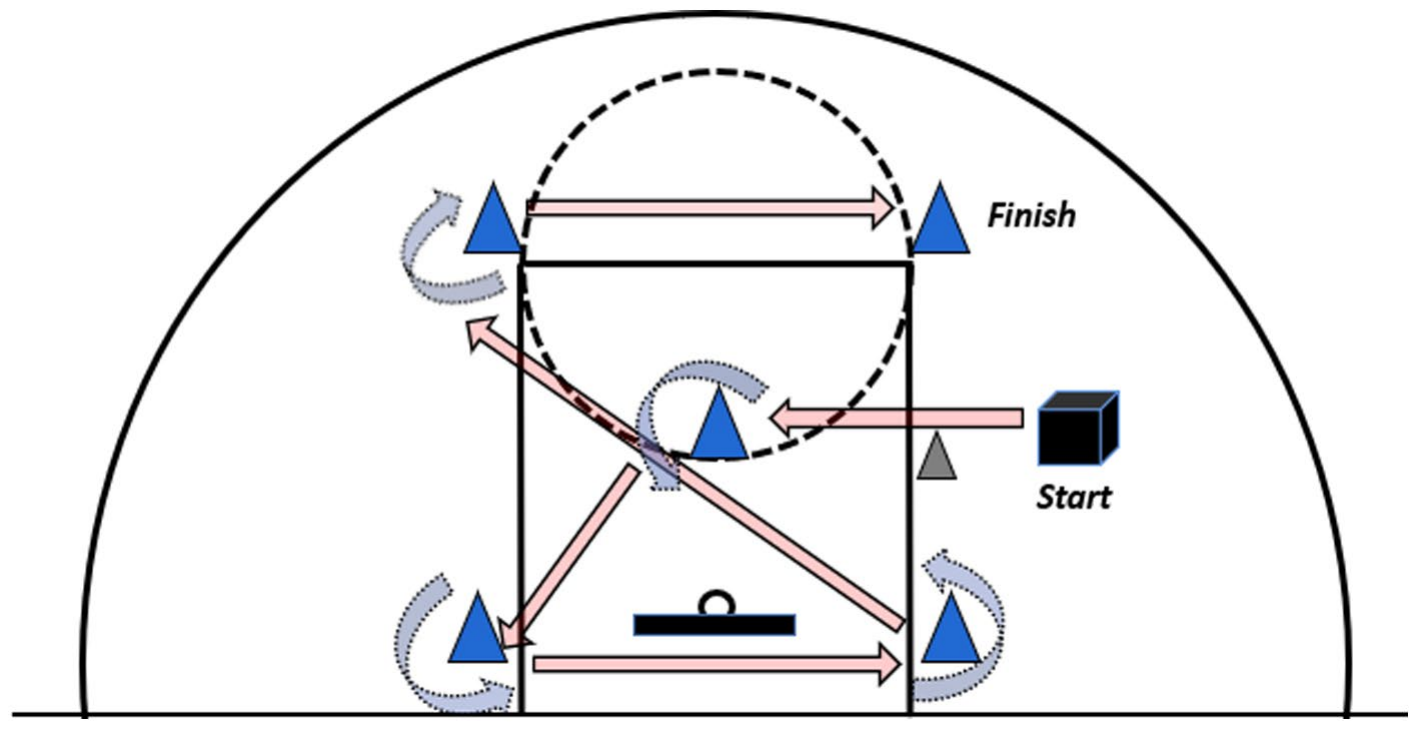
Obstacle dribbling test.

All participants completed three trials: the first served as a practice and familiarization phase, while the subsequent two were officially timed. A 3-min rest period was provided between each trial. Six cones were arranged on a standard basketball court to form the designated path shown in Figure 3. Participants were restricted to dribbling within the three-second zone. Upon hearing the command “Ready—Go,” the test began at Point A. Participants dribbled with their non-dominant hand to Point B, turned, switched to their dominant hand, and dribbled to Point C. They continued dribbling with the right hand to cone D. Then, switching to the left hand, they dribbled back to cone B. Dribbling with the right hand again, they proceeded to cone E, and continued with the right hand to the final cone F. This sequence followed the path BCDB (turnaround) EF. For the second official test, participants switched to starting with their dominant hand, reversing the path and hand-switching rules, while the rest of the procedure remained identical. The participants must maintain a low dribble and retain ball control throughout. Violations of the “three-second zone” rule, ball loss, or incorrect hand-switching resulted in an invalid attempt requiring retesting. The tester used a stopwatch to record the total time (in seconds) to complete the preset path. The combined time from both official tests constitutes the final score for this assessment.

#### 2.4.3 Defensive sliding test (DST)

**Figure 4:**
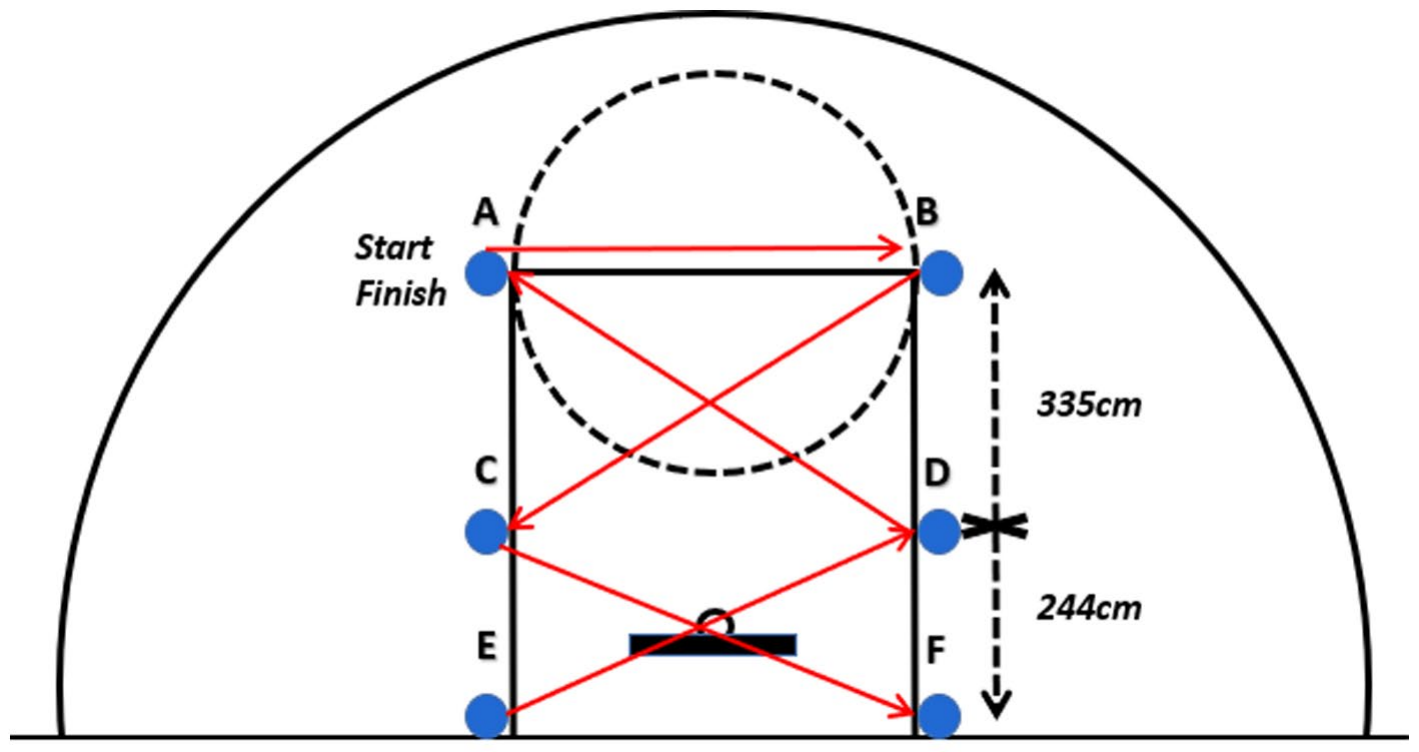
Defensive sliding test.

All participants completed three trials, the first served as a practice and familiarization phase, while the subsequent two were officially timed, official tests was taken as the final score. A 3-min rest period was scheduled between each trial. As illustrated in Figure 4, upon hearing the “Ready—Go” command, participants started from Point A, facing the center circle throughout the test. They moved to the right using a defensive shuffle stance. Upon reaching Point B, the participants touched the ground outside the line with their right hand, immediately turned, and moved leftward in a defensive shuffle to Point C, touching the ground outside the line with their left hand. They continued in this manner to Point D and other designated points until the shuttle run was completed. This formed a shuttle route starting from Point A, passing through Points B, C, D, and so on (Figure 4). Participants were strictly prohibited from crossing their legs or altering direction before touching the ground. Violations (e.g., crossing legs, premature turning, or failing to touch the ground) invalidated the attempt, requiring a restart from the starting point. However, the elapsed time was recorded as a penalty. The tester used a stopwatch to record the time required to complete the designated path (in seconds). The total time from two official attempts constitutes the final score for this test.

#### 2.4.4 Lay-up test (LUT)

**Figure 5:**
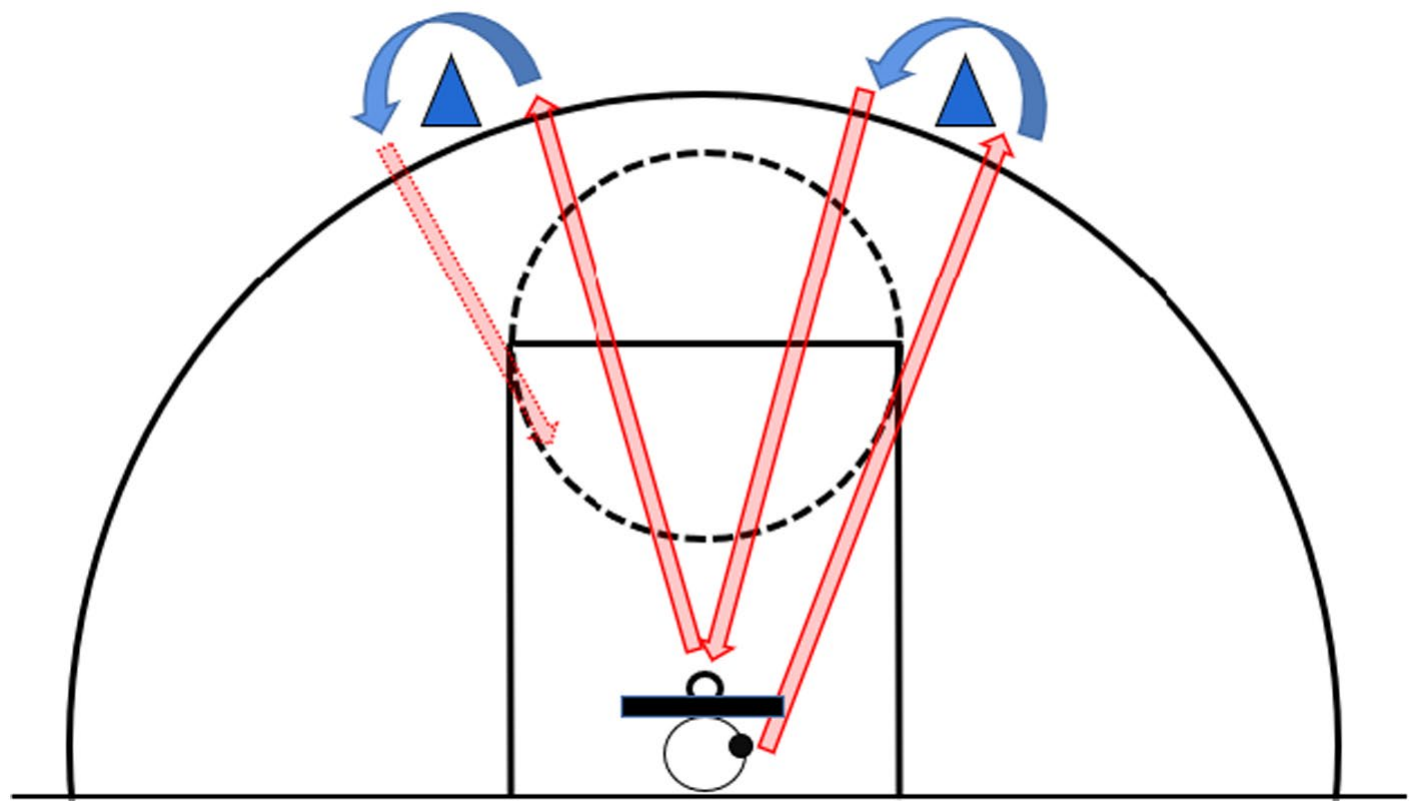
Lay-up test.

All participants completed three trials, the first served as a practice and familiarization phase, while the subsequent two were officially timed. A 3-min rest period was provided between each trial. Two cones were placed on the extended three-point line, spaced 6.25 meters apart (Figure 5). Upon hearing the “Ready—Go” command, participants positioned themselves behind the cones and could choose to jump from either the left or right side of the basket. They were required to complete as many dribble layups as possible within 2 min. After each layup attempt, the participant retrieved the rebound and dribbled back to the opposite cone to prepare for the next attempt. For each test, the total number of attempts and successful layups were recorded. The final score was the sum of the total attempts and successful layups from both timed trials.

#### 2.4.5 No ball maneuver running (NBMR)

**Figure 6:**
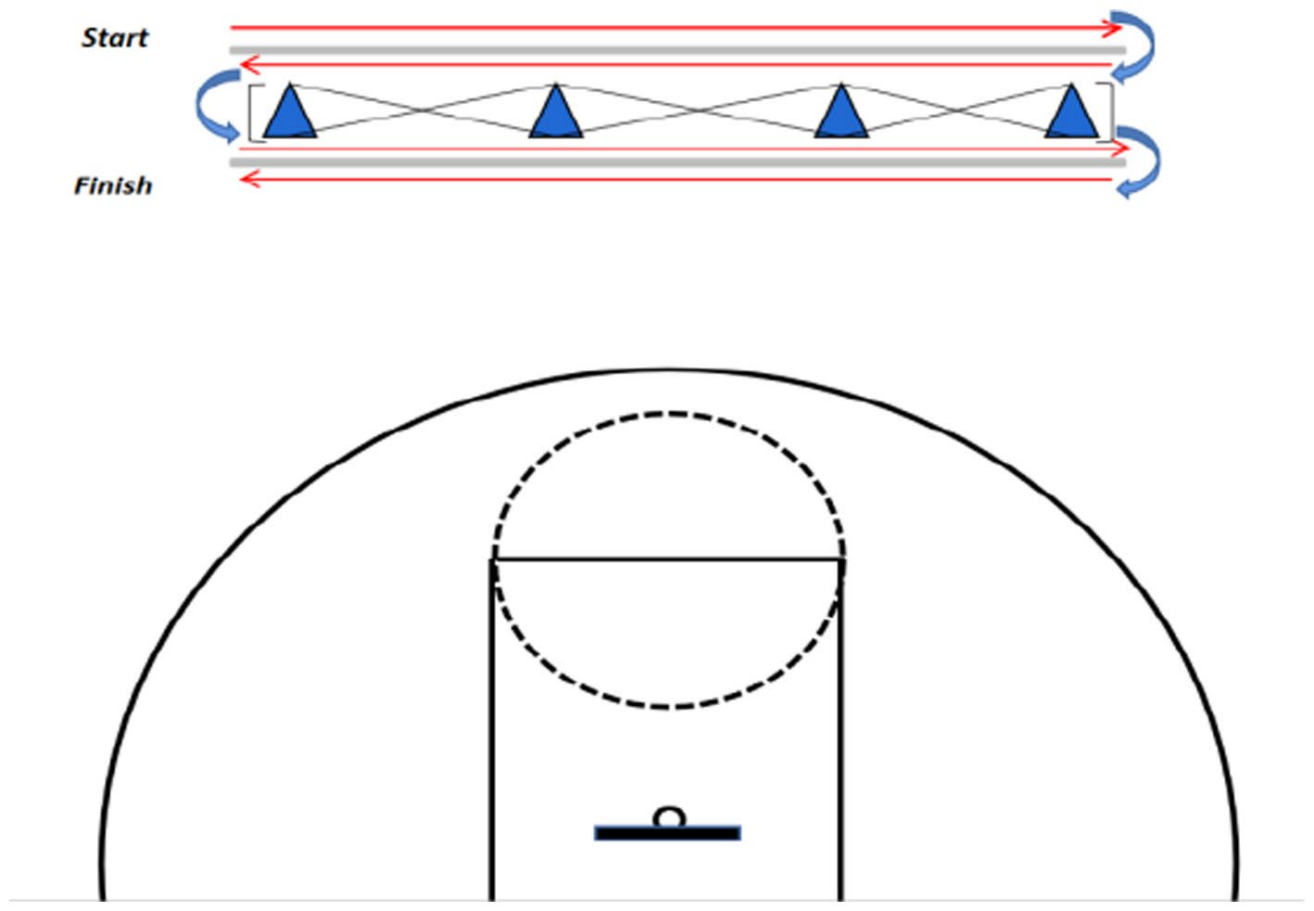
No ball maneuver running.

As shown in Figure 6, four cones spaced 3.33 meters apart were placed within a rectangular area measuring 10 meters long by 5 meters wide. All participants completed three trials, the first served as a practice and familiarization phase, while the subsequent two were officially timed. A 3-min rest period was provided between each trial. Upon hearing the “Ready—Go” command, the participants started from the first cone, sprinted at maximum speed to the last cone, then turned 180 degrees to return to the starting cone. This required the participants to shuttle back and forth between the cones, turning at the end cone and retracing the path to the starting cone. Upon reaching the terminal cone, participants turned 180 degrees again, passed through all cones, and returned to the starting cone. This test required one forward and one backward run, with the total time for both attempts recorded by experienced testers.

The selection of these five basketball skill assessments was guided by two principles: (1) coverage of core technical domains relevant to competitive basketball performance, and (2) differential sensitivity to psychological factors. Skills that are performed under time pressure or physical fatigue—such as the Speed and Accurate Shooting Test—are more likely to be influenced by psychological variables such as anxiety, attentional focus, and resilience, as these conditions approximate game-critical, high-stakes scenarios (e.g., game-winning shots under fatigue). In contrast, assessments of gross motor patterns such as the Defensive Sliding Test and No-Ball Maneuver Running reflect fundamental physical conditioning and locomotor efficiency, which are comparatively less sensitive to momentary psychological fluctuations. By including both categories of skills, the present study aimed to provide a comprehensive and ecologically valid assessment of the MAIC intervention’s effects across the psychological-technical performance spectrum.

### 2.5 Testing procedures and quality control

This study implemented mindfulness interventions in a quiet, well-lit, and spacious exercise laboratory. During each 60-min mindfulness training session, researchers first provided approximately 10 min of instruction on the mindfulness content to help athletes familiarize themselves with the training process and better complete the exercises. Attendance was tracked via sign-in sheets distributed before sessions. On-site researchers recorded participants’ focus levels by observing their adherence to audio instructions and frequency of distraction. Both control groups maintained their regular exercise routines and reported weekly activity duration, with all other psychological counseling or exercise strictly prohibited. Similarly, both experimental groups were prohibited from participating in any other psychological training or counseling sessions besides MAIC. Following the 8-week intervention, psychological performance assessments were conducted in a spacious, quiet lecture hall. All participants were instructed to truthfully complete documentation and attention tests, with strict prohibition against discussing the experiment or test content with others. Stimuli were presented via built-in E-prime 2.0 software on Lenovo laptops (15-inch, 1920 × 1080 resolution). Data collection was automated. Basketball technical skill assessments took place on a full-size 28 × 15 m standard basketball court, maintained in a quiet environment to minimize measurement error. Professional basketball coaches (hold a FIBA-issued Level B national basketball coach certification.) and assistant coaches observed and scored the tests. Participant order followed a Latin square design to reduce sequence and primacy/recency effects.

### 2.6 Statistical methods

Statistical analyses was performed using IBM SPSS Statistics (Version 26.0). Data are presented as mean ± standard deviation (M ± SD). The Shapiro–Wilk test was used to verify the normality of data distribution, and the Levene test was employed to assess homogeneity of variance. Independent samples *t*-tests and chi-square tests were used to assess heterogeneity between groups at baseline to ensure comparability. The core analysis employed a 2 (group: experimental vs. control) × 2 (level: elite vs. recreational) × 2 (time: pre-test vs. post-test) three-factor mixed design analysis of variance (ANOVA). Time served as the within-participant factor, while group and level were between-participant factors. If the three-way interaction (group ×level ×time) was significant, simple effects analysis was conducted to decompose the interaction. If only two-way interactions (e.g., time × group) were significant, simple effects analysis was performed. Multiple comparisons were adjusted using Bonferroni correction. Effect sizes were represented by partial Eta-squared, with 0.01, 0.06, and 0.14 denoting small, medium, and large effects, respectively. The significance level for all tests was set at *α* = 0.05.

## 3 Results

### 3.1 Baseline participant metrics

As shown in Table 2, independent samples t-tests and chi-square tests revealed no significant differences in pre-test indicators between elite basketball athletes mindfulness intervention group (EMIG) and elite basketball athletes control group (ECG), nor between recreational basketball mindfulness intervention group (RBMIG) and recreational basketball control group (RBCG). Homogeneity was well established, meeting the conditions for proceeding with the experiment.

**Table 2:** Descriptive statistics and homogeneity analysis of baseline indicators.

Indicators	Elite basketball athletes			
	EMIG	ECG	*t/X^2^*	*p*
Age	21.60 ± 1.64	21.35 ± 1.32	0.410	0.686
Sex (Female)	3(25.0%)	4(36.3%)	0.350	0.554
BMI	26.21 ± 4.35	26.10 ± 3.81	0.064	0.950
Weekly exercise duration	10.89 ± 1.23	11.14 ± 1.05	−0.531	0.601
Speed and accurate shooting test	44.98 ± 6.15	45.18 ± 5.56	−0.081	0.936
Obstacle dribbling test	21.23 ± 4.28	21.00 ± 3.99	0.135	0.894
Defensive sliding test	21.13 ± 2.07	21.02 ± 2.08	0.133	0.895
Lay-up test	12.95 ± 2.66	13.35 ± 2.76	−0.358	0.724
No ball maneuver running	31.78 ± 2.19	31.24 ± 2.33	0.57	0.575
STAI	49.83 ± 6.94	49.64 ± 6.65	0.069	0.945
BDI-II	17.25 ± 3.08	16.91 ± 2.59	0.288	0.776
SAT	33.98 ± 3.69	33.79 ± 3.24	0.127	0.900
CD-RISC	77.33 ± 5.21	77.45 ± 3.96	−0.063	0.950

Indicators	Recreational basketball players			
	RBMIG	RBCG	*t/X^2^*	*p*
Age	21.54 ± 2.08	21.37 ± 2.14	0.269	0.789
Sex (Female)	4(20.0%)	3(15%)	0.173	0.677
BMI	31.44 ± 3.89	31.35 ± 3.61	0.080	0.937
Weekly exercise duration	5.96 ± 1.18	6.05 ± 1.16	−0.230	0.819
Speed and accurate shooting test	36.94 ± 5.04	36.84 ± 4.73	0.061	0.951
Obstacle dribbling test	25.00 ± 2.46	24.96 ± 2.32	0.060	0.953
Defensive sliding test	28.40 ± 2.04	28.54 ± 2.07	−0.215	0.831
Lay-up test	9.47 ± 2.21	9.41 ± 2.23	0.093	0.927
No ball maneuver running	38.40 ± 2.04	38.54 ± 2.07	−0.215	0.831
STAI	31.99 ± 4.34	31.88 ± 4.03	0.079	0.937
BDI-II	12.44 ± 2.86	12.44 ± 2.89	−0.005	0.996
SAT	37.67 ± 3.01	38.21 ± 3.15	−0.559	0.579
CD-RISC	65.68 ± 7.56	65.56 ± 7.27	0.053	0.958

EMIG, Elite Basketball athletes Mindfulness Intervention Group; ECG, Elite Basketball athletes Control Group; RBCG, Recreational basketball control group; RBMIG, Recreational basketball mindfulness intervention group.

### 3.2 Three-factor mixed ANOVA results

**Figure 7:**
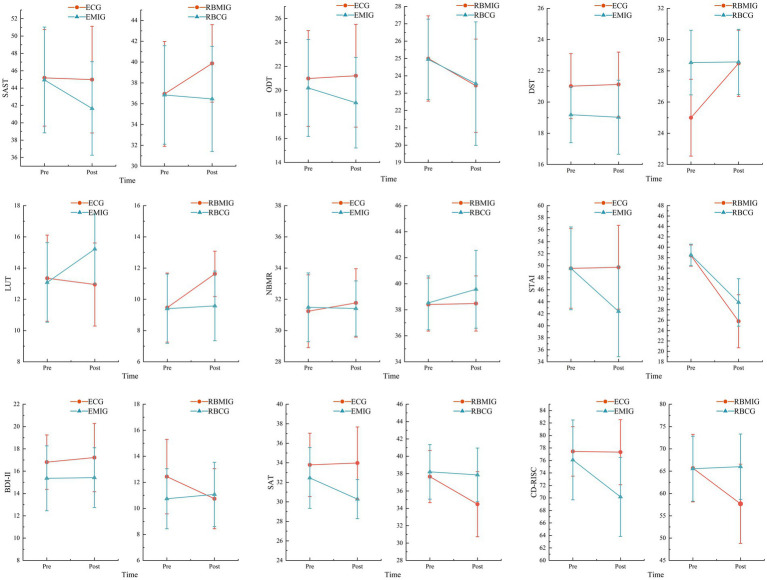
Visualization results of multiple groups before and after MAIC.

This study employed a 2 × 2 × 2 repeated measures ANOVA and found that the 8-week MAIC significantly impacted basketball players’ psychological and technical performance, with multiple core indicators exhibiting significant interaction effects. As shown in Table 3, in the Speed and Accuracy Shooting Test, a significant triple interaction was identified (group × level × time, *p* = 0.007, $n_{p}^{2}$ =0.126), where the recreational experimental group’s scores significantly increased post-intervention (*p* = 0.014), while the elite experimental group’s scores decreased. This reveals a significant moderating effect of skill level on the impact of mindfulness intervention on fundamental technical performance. Both the Layup Test and anxiety levels showed significant time-by-group interactions (*p* = 0.002, $n_{p}^{2}$ = 0.166 and 0.275, respectively), confirming that mindfulness intervention effectively enhances layup stability and psychological regulation across both skill levels. Notably, the experimental group demonstrated a significantly greater reduction in anxiety scores compared to the control group (95% CI [−5.455, −2.544]). Furthermore, the significant time-by-level interaction effect in the Obstacle Dribbling Test (*p* < 0.001, $n_{p}^{2}$ =0.307) reflects natural variations in skill development across player levels during the training cycle. Overall, recreational players demonstrated more comprehensive gains across technical and psychological dimensions, whereas elite athletes primarily benefited from enhanced psychological resilience and sustained technical stability under high-pressure conditions. Figure 7 displays the results of this study visually, allowing for an intuitive observation of the differences in MAIC effectiveness across participants through line interactions and slopes.

**Table 3:** Results of the 2 × 2 × 2 mixed design ANOVA.

Indicators	Testing point	ECG	EMIG	RBCG	RBMIG	Interaction item	95% CI	*p*	$n_{p}^{2}$
SAST	Pre	45.18 ± 5.56	44.94 ± 6.09	36.84 ± 4.73	36.94 ± 5.04	Group*Level*Time	[−1.616, 1.107]	0.007^*^	0.126
	Post	44.98 ± 6.15	41.65 ± 5.39	36.46 ± 5.05	39.87 ± 3.72	Group*Time		0.393	0.013
						Level*Time		0.004^*^	0.145
ODT	Pre	21.00 ± 3.99	20.21 ± 4.04	24.95 ± 2.32	25.00 ± 2.46	Group*Level*Time	[−2.477, −0.531]	0.595	0.005
	Post	21.23 ± 4.28	18.98 ± 3.77	23.55 ± 3.56	23.43 ± 2.69	Group*Time		0.382	0.014
						Level*Time		<0.001^*^	0.307
DST	Pre	21.02 ± 2.08	19.19 ± 1.79	28.53 ± 2.07	28.39 ± 2.14	Group*Level*Time	[−1.567, −0.332]	0.717	0.002
	Post	21.13 ± 2.07	19.03 ± 2.37	28.57 ± 2.09	28.48 ± 2.52	Group*Time		0.969	0.000
						Level*Time		0.246	0.024
LUT	Pre	13.35 ± 2.76	13.08 ± 2.55	9.40 ± 2.22	9.47 ± 2.21	Group*Level*Time	[0.309, 1.858]	0.703	0.003
	Post	12.95 ± 2.66	15.22 ± 2.24	9.58 ± 2.23	11.63 ± 1.45	Group*Time		0.002^*^	0.166
						Level*Time		0.003^*^	0.150
NBMR	Pre	31.24 ± 2.33	31.49 ± 2.20	38.53 ± 2.07	38.40 ± 2.04	Group*Level*Time	[−0.732, 0.741]	0.572	0.006
	Post	31.77 ± 2.19	31.41 ± 1.77	39.57 ± 2.99	38.48 ± 2.12	Group*Time		0.73	0.002
						Level*Time		0.392	0.013
STAI	Pre	49.55 ± 6.64	49.56 ± 6.89	38.53 ± 2.07	38.40 ± 2.04	Group*Level*Time	[−5.455, −2.544]	0.171	0.034
	Post	49.74 ± 6.97	42.39 ± 7.54	29.41 ± 4.56	25.79 ± 5.10	Group*Time		<0.001^*^	0.275
						Level*Time		<0.001^*^	0.349
BDI-II	Pre	16.81 ± 2.44	15.36 ± 2.91	10.74 ± 2.31	12.44 ± 2.86	Group*Level*Time	[−2.163, −1.053]	0.914	0.000
	Post	17.22 ± 3.06	15.42 ± 2.68	11.07 ± 2.47	10.74 ± 2.31	Group*Time		0.411	0.012
						Level*Time		0.018^*^	0.097
SAT	Pre	33.79 ± 3.24	32.45 ± 3.12	38.21 ± 3.15	37.66 ± 3.01	Group*Level*Time	[−3.100, −1.186]	0.753	0.002
	Post	33.98 ± 3.69	30.28 ± 2.01	37.86 ± 3.09	34.48 ± 3.75	Group*Time		0.004^*^	0.140
						Level*Time		0.043^*^	0.072
CD-RISC	Pre	77.45 ± 3.96	76.09 ± 6.39	65.55 ± 7.27	65.68 ± 7.56	Group*Level*Time	[−6.161, −1.874]	0.604	0.005
	Post	77.33 ± 5.21	70.17 ± 6.32	66.01 ± 7.32	57.69 ± 8.87	Group*Time		<0.001^*^	0.246
						Level*Time		0.072	0.058

SAST, Speed and accurate shooting test; ODT, Obstacle dribbling test; DST, Defensive sliding test; LUT, Lay-up test; NBMR, No ball maneuver running; ECG, Elite control group; EMIG, Elite mindfulness intervention group; RBCG, Recreational basketball control group; RBMIG, Recreational basketball mindfulness intervention group; *p < 0.05.

## 4 Discussion

This study examined the effects of an 8-week MAIC program on psychological and technical skill-related metrics among elite basketball athletes and college basketball players, while also investigating differences in improvement across various metrics following MAIC among heterogeneous groups. Results indicate significant differences in benefits across skill levels: elite athletes primarily demonstrated significant improvements in anxiety, resilience and layup performance, while recreational players exhibited broader gains including shooting accuracy, layup proficiency, anxiety alleviation, attention improvement, and resilience enhancement. The three-way interaction effect indicates that MAIC yield significantly greater effects for recreational players than for elite players. This study aligns with previous meta-analytic evidence on mindfulness interventions and indicates how athletes’ skill levels and psychological states serve as crucial moderating factors for MBIs (Bondar et al., 2024; Cao et al., 2024; Cioffi et al., 2025; Goisbault et al., 2022; Su et al., 2019).

Among elite athletes, MAIC led to significantly reduced anxiety levels alongside markedly enhanced resilience, with attention showing marginal improvement. These psychological benefits align with existing research findings, demonstrating that mindfulness training positively impacts the mental state of high-level athletes. This includes boosting mindfulness levels, resilience, and flow states while alleviating anxiety, depression, and mental fatigue (Zhang et al., 2025). Koop and Jooste (2023) demonstrated that higher mindfulness traits inhibit irrational performance beliefs and uncertainty intolerance among elite female basketball players, aligning with the core components of the MAIC program—“present-moment awareness and cognitive flexibility.” Preliminary research found mindfulness training reduced athletes’ depression, perceived stress, and anxiety while enhancing self-esteem and the ability to cope positively with high-pressure environments (Stoyanova et al., 2025). Notably, the intervention’s impact on layup performance suggests a transfer of psychological enhancement to motor execution, partially supporting embodied cognition theory. This posits that mindfulness-induced reductions in cognitive interference free up attentional resources, enabling smoother movement—particularly in basketball-specific tasks like layups requiring rapid decision-making and kinesthetic awareness (Bondar et al., 2024). However, the absence of significant changes in other technical skills (e.g., shooting, dribbling) may reflect a ceiling effect due to elite athletes’ baseline proficiency (Reinebo et al., 2024). This pattern suggests that mindfulness interventions may be more effective at refining psychological factors rather than fundamentally transforming foundational basketball skills in highly skilled athletes (Ptáček et al., 2024).

Compared to elite athletes, recreational basketball players derived broader benefits from MAIC, demonstrating significant improvements in shooting accuracy, layup performance, anxiety, attention, and resilience. These more comprehensive effects corroborate previous findings that MBIs yield greater benefits among non-elite athletes, potentially due to their lower baseline psychological and technical levels (Yang et al., 2025). Wang demonstrated that a 7-week mindfulness intervention significantly enhanced mindfulness levels, acceptance, attention, and shooting performance among male college basketball players, aligning with the present study (Wang et al., 2023b). Cao indicated that brief mindfulness interventions alleviate mental fatigue in basketball tactics, benefiting decision-making and execution—a finding consistent with reduced depression and anxiety among recreational players (Cao et al., 2024). A plausible mechanism for these performance benefits may stem from MAIC’s integration of cultural and regional elements relevant to participants, enhancing engagement and adherence among recreational basketball players. Additionally, the lower intrinsic motivation typically observed in recreational players compared to elite athletes may partially contribute to these effects (Kanaujia et al., 2023). This study observed that MAIC showed no intervention effects on defensive sliding and agility skills. This may indicate that MBIs may prioritize tasks based on fine motor skills rather than gross motor skills, indirectly corroborating the emphasis on the role of mindfulness-based stress reduction protocols in enhancing flow states and competitive performance among basketball players (Cioffi et al., *2025*).

Results from the comparative covariance analysis revealed that, after controlling for baseline, recreational players demonstrated greater improvement than elite athletes in shooting, dribbling, anxiety, and depression, indicating that skill level may serve as a moderating factor. This observed difference supports prior findings of a “novice advantage” in MBIs, where hobbyists exhibit higher baseline performance variability, thereby allowing greater room for improvement (Xie et al., 2025). Theoretically, MAIC reinforces the embodied cognition framework, where mental states form positive feedback loops through the body, amplified more readily in recreational players (Kim and Kim, 2021). Goisbault et al. (2022) suggests that programs integrating mindfulness and acceptance, mediated by personality traits, enhance performance satisfaction and emotional regulation—further explaining the observed skill-level differences. Practically, this study provides evidence-based guidance for athletes at different levels: elite groups can utilize MAIC for psychological optimization, while recreational groups can employ it for comprehensive enhancement (Tingaz, 2025).

Despite valuable insights, this study has several limitations. (1) The relatively small sample size may have limited statistical power and increased the risk of Type II errors, particularly for marginally significant results like attention metrics. (2) The intervention lasted only 8 weeks without follow-up measurements, potentially underestimating long-term maintenance effects. (3) Participants primarily came from a Chinese cultural context; while the cultural adaptation of the MAIC protocol enhanced applicability, it may limit generalizability to other cultures or diverse populations. (4) Self-report measures may be susceptible to social desirability bias. (5) The control group received only routine training without placebo intervention, potentially introducing expectancy effect bias. (6) Although the basketball-specific skill test has been standardized, it does not incorporate real game scenarios, potentially underestimating the actual impact of MBIs under high-pressure conditions. (7) Existing literature does not support the external validity and reliability of basketball skill tests, and caution is advised when applying this study’s testing protocol to different populations. (8) Due to the inherent nature of MBIs, blinding of participants and intervention facilitators was not feasible. Assessors were also not blinded to group allocation during data collection. (9) A related limitation concerns the potential heterogeneity of occupational backgrounds within the recreational basketball enthusiast group. While we attributed the larger intervention-induced gains among recreational players primarily to their lower baseline levels of psychological resilience, attentional capacity, and stress tolerance relative to elite athletes, this interpretation may be complicated by occupational factors. Recreational enthusiasts who are employed in high-stress, cognitively demanding professions may already possess robust psychological coping resources comparable to those of trained athletes. (10) This experiment relied solely on manual timing for some skill-related tests and did not use more precise timing instruments, so there may be some limitations in terms of accuracy. (11) In this study, the SAT is essentially a training method; its validity and reliability for assessing attention levels remain to be tested. These limitations may introduce performance and detection bias, and should be considered when interpreting the findings. Future research should validate these findings through larger, multicenter randomized controlled trials incorporating diverse samples across gender, age, and cultural backgrounds to enhance external validity. Long-term follow-ups should assess intervention persistence and explore dose–response relationships, such as optimizing intervention frequency and duration. Integrating neuroimaging methods (e.g., fMRI) could reveal MBIs’ mechanistic effects on attention networks and emotion-regulating brain regions.

## 5 Conclusion

MAIC can have a significant impact on a range of psychological and skill-related indicators; at the same time, recreational players stand to gain more comprehensive benefits from MAIC than elite basketball players.

## Data Availability

The original contributions presented in the study are included in the article/Supplementary material, further inquiries can be directed to the corresponding authors.
