# Supplementary material for: Enhancing psychological performance and basketball skills: a comparative study of elite athletes and college recreational players after an 8-week mindfulness intervention
Source: Front Psychol. 2026 Apr 22;17:1794656. doi: 10.3389/fpsyg.2026.1794656 (PMC13144095; doi:10.3389/fpsyg.2026.1794656)
Supplement: Supplementary file 1 [file Data_Sheet_1.PDF]

Supplementary Document 1: Detailed Report on the MAIC Procedure	
1.mindful breathing exercise：	As a basic introductory exercise, its core goal is to strengthen the mindfulness abilities in the dimensions of “observing” and “non-judging.” By guiding athletes to focus on the natural rhythm of breathing (such as the flow of air through the nostrils and the rise and fall of the abdomen), it helps them quickly bring their attention back to the present moment when distracted by random thoughts, reducing excessive worries about past mistakes or future outcomes. This exercise lays the foundation for maintaining concentration in subsequent complex scenarios, and is particularly suitable for stages of action execution that require a high degree of concentration, such as free throws.
2.Concentration exercise:	By focusing on a single object, it deliberately trains athletes’ ability to resist environmental interference, directly corresponding to the “acting with awareness” dimension. In basketball free-throw scenarios, athletes often face external stimuli such as audience noise and opponent interference. This exercise can enhance their sustained attention to the target (the basket) and reduce the disruption of irrelevant information to the coherence of movements.
3.Body scan exercise:	It requires athletes to perceive physical sensations part by part (from toes to the top of the head), focusing on cultivating “observing” and “describing” abilities. For free throws, accurate physical perception (such as wrist force and knee bending angle) is crucial for standardizing movements. This exercise can help athletes identify physical tension signals under stress (such as stiff shoulders and neck) and restore the best state through active adjustment.
4.Mindful fruit eating/slow-motion drinking exercise:	By slowing down daily actions and carefully perceiving sensory experiences (such as the texture of fruits and the feel of water flowing through the throat), it strengthens the “acting with awareness” and “non-reacting” dimensions. Such exercises simulate the need to “break down movement steps” during free throws, prompting athletes to get rid of the automatic inertia of movements and avoid movement deformities caused by stress (such as hasty shots).
5.Mindful walking exercise:	Maintaining awareness of foot landing and body weight transfer during movement, combining the state of mindfulness with dynamic actions, is closer to the actual scene of basketball. Its role is to train athletes to maintain concentration during physical activities, preventing distractions during movement (such as outside movements) from affecting subsequent free-throw preparations.

6.Mindful meditation exercise:	Through open awareness (such as accepting all thoughts without getting involved), it deepens the abilities of "non-judging" and "non-reacting." This is directly related to emotional management under stress - when athletes have self-doubt due to mistakes, this exercise can help them view emotions with a neutral attitude and prevent the spread of negative thoughts from affecting the next free throw.
7.Mindful number exercise:	It trains the stability of attention through counting down numbers or memorizing number sequences, and introduces slight interference (such as occasional sound prompts) to simulate sudden stress in competitions. This exercise specifically improves athletes' ability to quickly recover after their attention is briefly interrupted, which is highly consistent with the scenario of dealing with sudden cheers from the audience during free throws.
Note：These exercises are not randomly selected; instead, they progressively cover the five core dimensions of mindfulness through step-by-step training (observation, description, conscious action, non-judgment, and non-reactivity). They also closely integrate the movement characteristics of basketball free throws and the demands of pressure scenarios, ultimately achieving a transition from foundational mental skills to specific performance outcomes.	
